# Multidisciplinary Intervention After Dental Trauma in a Young Patient During Sports Practice: A Case Report

**DOI:** 10.1155/crid/6704067

**Published:** 2026-05-22

**Authors:** Alice Corrêa Silva-Sousa, Rafael Verardino Camargo, Lautaro Gallardo Altube, André Pitondo-Silva, Fernanda Gonçalves Basso, Jardel Francisco Mazzi-Chaves, Manoel Damião Sousa-Neto, Francisco Wanderley Garcia de Paula-Silva

**Affiliations:** ^1^ Department of Restorative Dentistry, School of Dentistry of Ribeirão Preto, University of São Paulo (USP), Ribeirão Preto, São Paulo, Brazil, usp.br; ^2^ Postgraduate Program in Dentistry, School of Dentistry, University of Ribeirão Preto, Ribeirão Preto, São Paulo, Brazil, unaerp.br

**Keywords:** dental trauma, paraendodontic surgery, sport injury

## Abstract

This case report describes the multidisciplinary management of a young patient after sports‐related dental trauma. A 13‐year‐old girl was referred to the endodontics service after initial emergency care for evaluation of persistent apical periodontitis associated with the permanent maxillary left central incisor (20). The treatment plan, based on CBCT images, included the removal of the orthodontic intracanal wire, which had been used for orthodontic traction, followed by adhesive restoration of Tooth 21 and paraendodontic surgical intervention performed under magnification. During surgery, biological material was collected using sterile paper cones to obtain biofilm material. Granulation tissue was curetted and processed for histological analysis. It was possible to observe the presence of dense connective tissue associated with diffuse inflammatory infiltrate, classified as a periapical granuloma. Microbiological analysis identified *Pseudomonas aeruginosa* in the clinical sample obtained during paraendodontic surgery. Six‐month CBCT follow‐up demonstrated periapical bone healing. This case highlights the importance of long‐term multidisciplinary management after dental trauma in young patients.

## 1. Introduction

Managing traumatic dental injuries (TDIs) presents significant challenges for dentists. Several factors affect the outcome of treatment, including the time elapsed between the injury and initial intervention, the presence or absence of contamination at the injury site, and the severity of the TDI [1, 2]. Common complications include pulp necrosis, root canal obliteration, root resorption, and ankylosis [2–4]. Additionally, tooth loss resulting from the trauma is frequently observed [3]. Therefore, effective clinical management of teeth with a history of TDI necessitates a comprehensive understanding of the etiology, accurate diagnosis, meticulous treatment planning, and informed prognosis of these injuries [5].

Dental trauma during sports activities is frequently encountered and can lead to considerable damage to teeth and adjacent structures [6]. Contact sports, such as jiu‐jitsu, present a heightened risk of dental injuries due to falls, direct impacts, or collisions. These injuries not only compromise dental aesthetics and function but often require intricate dental procedures, including endodontic and surgical interventions, to restore oral health and prevent future complications, such as tooth loss [6]. Effective management of these cases necessitates a multidisciplinary approach that integrates restorative dentistry, endodontics, and, when necessary, surgical treatment. This highlights the critical importance of prevention and prompt intervention to reduce long‐term consequences [7].

Apical periodontitis might emerge as a sequela of dental trauma, and it is documented in the literature as an inflammatory and immune response condition predominantly induced by polymicrobial infection of the dental pulp and root canals [8, 9]. It is characterized by a mixed inflammatory infiltrate that includes neutrophils, T and B lymphocytes, plasma cells, macrophages, and dendritic cells, with the prevalence of these cells varying according to the stage of the disease [10, 11]. This inflammatory process affects and damages the surrounding tissues, leading to the destruction of the periodontal ligament, cementum, and alveolar bone around the root apex [8–10], primarily due to osteoclastic activity [8, 9, 12].

Cone beam computed tomography (CBCT) has become extensively utilized in endodontics, especially for diagnosing apical periodontitis, due to its superior sensitivity and accuracy compared to traditional periapical radiographs [13]. CBCT enables volumetric analysis, localization, and assessment of the extent of periapical lesions through reconstruction and visualization in various orthogonal planes [13, 14]. Additionally, CBCT is invaluable in cases of dental trauma, facilitating precise clinical planning for complex situations and aiding in diagnosing conditions such as root resorption and coronal fractures. This case report details the multidisciplinary planning and treatment of a young patient′s upper central incisor, which presented persistent apical periodontitis following endodontic treatment due to dental trauma.

## 2. Case Report

A 13‐year‐old female patient (G.G.G.) was referred to the endodontics clinic at the School of Dentistry of Ribeirão Preto, University of São Paulo (FORP/USP), after dental trauma and initial treatment for evaluation of persistent apical periodontitis. During the anamnesis, satisfactory general health was noted, with no medical history of illness. The patient reported that the trauma occurred due to a fall while practicing the sport of jiu‐jitsu. The patient′s grandmother, who was responsible for the minor, signed the consent form authorizing treatment. The case was approved by the institutional Research Ethics Committee (CAAE: 45617721.3.0000.5419) and is reported in accordance with the guidelines of the Preferred Reporting Items for Case reports in Endodontics (PRICE) [15].

Clinical examination revealed an orthodontic intracanal wire for the traction of the permanent maxillary left central incisor (20) after a crown–root fracture. Radiographic examination showed a radiolucent area suggestive of a periapical lesion as well as evidence of previous endodontic treatment. The patient exhibited significant gingival inflammation. The retainer was removed under magnification using an ultrasonic scaler. Considering the patient′s age and remaining tooth structure, a fully adhesive restorative approach was selected to avoid indirect restorations.

**Figure 1:**
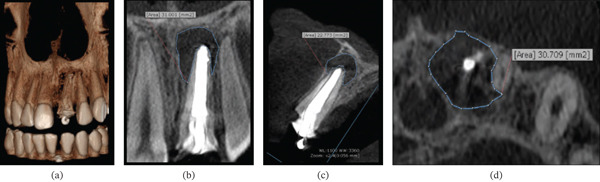
Diagnostic examination and planning of paraendodontic surgery using cone beam computed tomography. (a) 3D reconstructions, (b) coronal, (c) sagittal, and (d) axial views showing the quality of previous endodontic treatment, the location of the periapical lesion at the apical level, and the extent of the lesion, highlighting the breach of the vestibular bone plate.

A CBCT scan (EAGLE 3D, Dabi Atlante) was obtained in accordance with the American Association of Endodontists′ guidelines [16, 17]. The scan was acquired in high resolution (HD mode) with a 5 × 5 mm FOV, operating at 85 kV and 6.3 mA, with a 25.5‐s exposure and an isotropic voxel of 0.100 mm^3^. The images were then automatically reconstructed using the On‐Demand 3D program (Cybermed Inc., Tustin, CA, United States) (Figure 1).

The CBCT data were used for diagnosis and paraendodontic surgical planning, considering the following aspects: shape (circularity and form factor) [18, 19], lesion location and spatial position, extent in relation to adjacent teeth and anatomical structures, the relationship between the tooth and periapical lesion, lesion area (square millimeter), density of the lesion and surrounding alveolar bone, and the degree of bone and root resorption.

After the guardian signed the informed consent form, acknowledging the risks and benefits of the treatment, an appointment was scheduled for tooth restoration and subsequent paraendodontic surgery.

### 2.1. Restorative Procedures

After prophylaxis and shade selection using the homologous tooth, the remaining dental substrate was regularized using a round diamond bur. Due to significant loss of tooth structure, a clamp was avoided, and relative isolation was achieved using a #00 retraction cord (Ultrapak), a lip retractor, and cotton rolls to maintain a moisture‐free field.

The restorative materials were used in the following sequence: Ribbond containment and reinforcement ribbon, Brilliant NG composite resins (Coltene), Filtek Z250 XT (3M), Adper Single Bond 2 adhesive (3M), and 37% phosphoric acid (Biodinâmica). Two pieces of Ribbond tape were cut to prepare a splint between the adjacent teeth: one extending between the abutment teeth (right central incisor and left lateral incisor) and another intended to reinforce the permanent maxillary right central incisor. The material was handled according to the manufacturer′s instructions.

**Figure 2:**
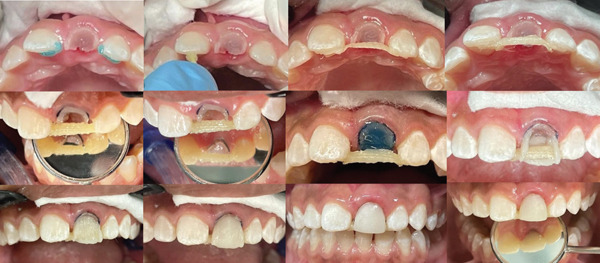
Sequence of the fully adhesive restorative procedure.

The palatal and proximal enamel surfaces (mesial of permanent maxillary right central incisor and distal of permanent maxillary left lateral incisor) were conditioned with 37% phosphoric acid for 15 s, rinsed, and dried. Adper Single Bond 2 adhesive was applied and light‐cured. A layer of composite resin (Filtek Z250 XT) was applied to these surfaces to embed the first precut Ribbond tape, which was soaked in the adhesive and adjusted onto the treated surfaces. Excess resin was removed, and the assembly was light‐cured. The same steps were repeated for the second tape (Figure 2).

With the splint adhered, the remaining tooth surface was treated (acid conditioning + adhesive application), and the palatal surface was shaped using a transparent strip as a guide. The proximal and vestibular surfaces were then reconstructed freehand with the previously selected composite resin colors (Figure 2).

After stratification and light curing, occlusal adjustment, finishing, and polishing were performed using a No. 12 scalpel blade, multilaminated bur, polishing discs, abrasive rubber points, interproximal polishing strips, and a felt disc with high‐gloss diamond paste.

Following the restorative phase, the patient′s functional and aesthetic rehabilitation was closely monitored. During the follow‐up period, the patient reported a complete return to normal activities, specifically regarding masticatory efficiency, phonetics, and smiling confidence. There were no reports of discomfort or functional limitations.

### 2.2. Endodontic Surgery

**Figure 3:**
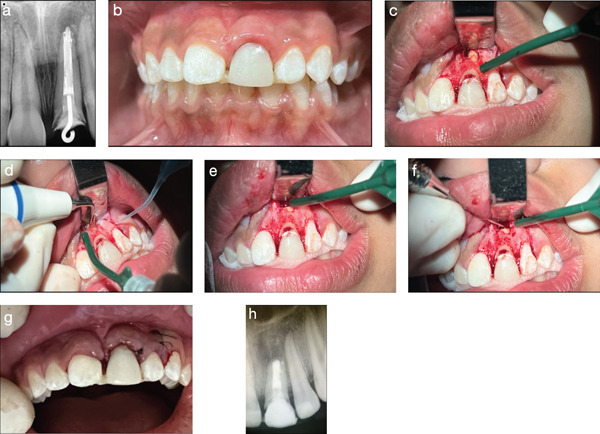
Moments of the paraendodontic surgery. (a) Preoperative radiograph. (b) Initial view. (c–e) Access for curettage, periapical lesion removal, and apicoectomy. (f) Retrofilling procedure. (g) Suturing of the surgical wound. (h) Postoperative radiograph.

Ten days postrestoration, paraendodontic surgery was performed under local anesthesia and aseptic conditions, using a surgical microscope (Figure 3) [20]. A relaxing incision was made distal to the permanent maxillary left lateral incisor, followed by dissection of the papilla from the mesial of the permanent maxillary right central incisor to the distal of the permanent maxillary left lateral incisor. The gingival tissue was detached using a Molt 1 dissector, exposing the surgical area with bone fenestration (Figure 3c).

Osteotomy was performed with a spherical FG 1014 diamond bur under constant irrigation. Granulation tissue was curetted and placed in 10% buffered formalin for histological and immunohistochemical processing. Sterile paper points were used to collect biofilm material from the apical region, with samples transferred to brain heart infusion (BHI) broth and thioglycolate broth for microbiological analysis [21].

An apicoectomy was conducted using a Bladesonic ultrasonic insert, followed by retrofilling with an ultrasonic insert P1—surgical standard (Figure 3d,e). The canal was dried with absorbent paper points and filled with Bio‐C Repair bioceramic cement (Angelus, Londrina, PR, Brazil). Radiographic verification confirmed the quality of the retrofilling (Figure 3f).

The surgical site was curetted to induce clot formation, and the gingival tissue was repositioned using Gerald forceps. Suturing was done with 5/0 sutures, assisted by Corn suture forceps and a Castroviejo needle holder (Figure 3g).

Postsurgery, the patient was prescribed analgesics and anti‐inflammatory medication. Ten days later, the patient returned for suture removal and radiographic follow‐up. A mouthguard was also fabricated and delivered for use during sports activities.

### 2.3. Histopathological Findings

**Figure 4:**
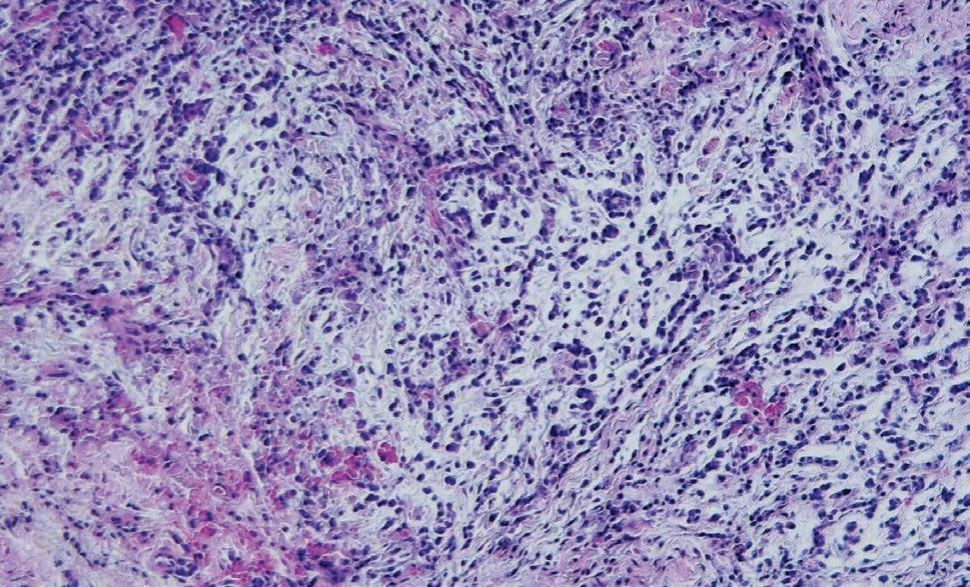
Photomicrographs of histological sections from representative samples of periapical granuloma lesions, subjected to immunohistochemistry (20x). Dense connective tissue with intense diffuse inflammatory infiltrate (PG).

The sample of periapical lesions was fixed in 10% buffered formalin and processed according to conventional histological procedures, followed by embedding in paraffin blocks. Subsequently, 5 *μ*m histological sections were obtained using a manual microtome and stained with hematoxylin and eosin (H&E). Histopathological analysis of the H&E‐stained sections showed dense connective tissue associated with a diffuse inflammatory infiltrate, and the lesion was diagnosed as a periapical granuloma (Figure 4).

### 2.4. Microbiological Findings

Microbiological analysis was performed to identify microorganisms involved in the apical periodontitis. The paper cones containing the collected material were incubated in two conditions: BHI broth at 37°C in a standard incubator and thioglycolate broth in an anaerobic chamber with 10% CO_2_.

For bacterial isolation from the BHI broth, 10 *μ*L of culture was streaked onto three media: m‐Enterococcus Agar (Acumedia), Müeller–Hinton Agar (Acumedia), and MacConkey Agar (Acumedia). The plates were incubated at 37°C under aerobic conditions. After 24 h, growth was observed only on the Müeller–Hinton Agar. The m‐Enterococcus Agar and MacConkey Agar plates showed no growth after 72 h. The two different colonies on Müeller–Hinton Agar were further isolated, incubated, and preserved in BHI broth with 15% glycerol at −80°C.

The isolates were identified by matrix‐assisted laser desorption/ionization time‐of‐flight mass spectrometry (MALDI‐TOF; Bruker Daltonics). Spectral peaks were compared with a reference library, with score values ≥ 1.7 being considered indicative of reliable genus and species identification [22]. The analysis identified *Pseudomonas aeruginosa* in the clinical sample.

### 2.5. Follow‐Up

**Figure 5:**
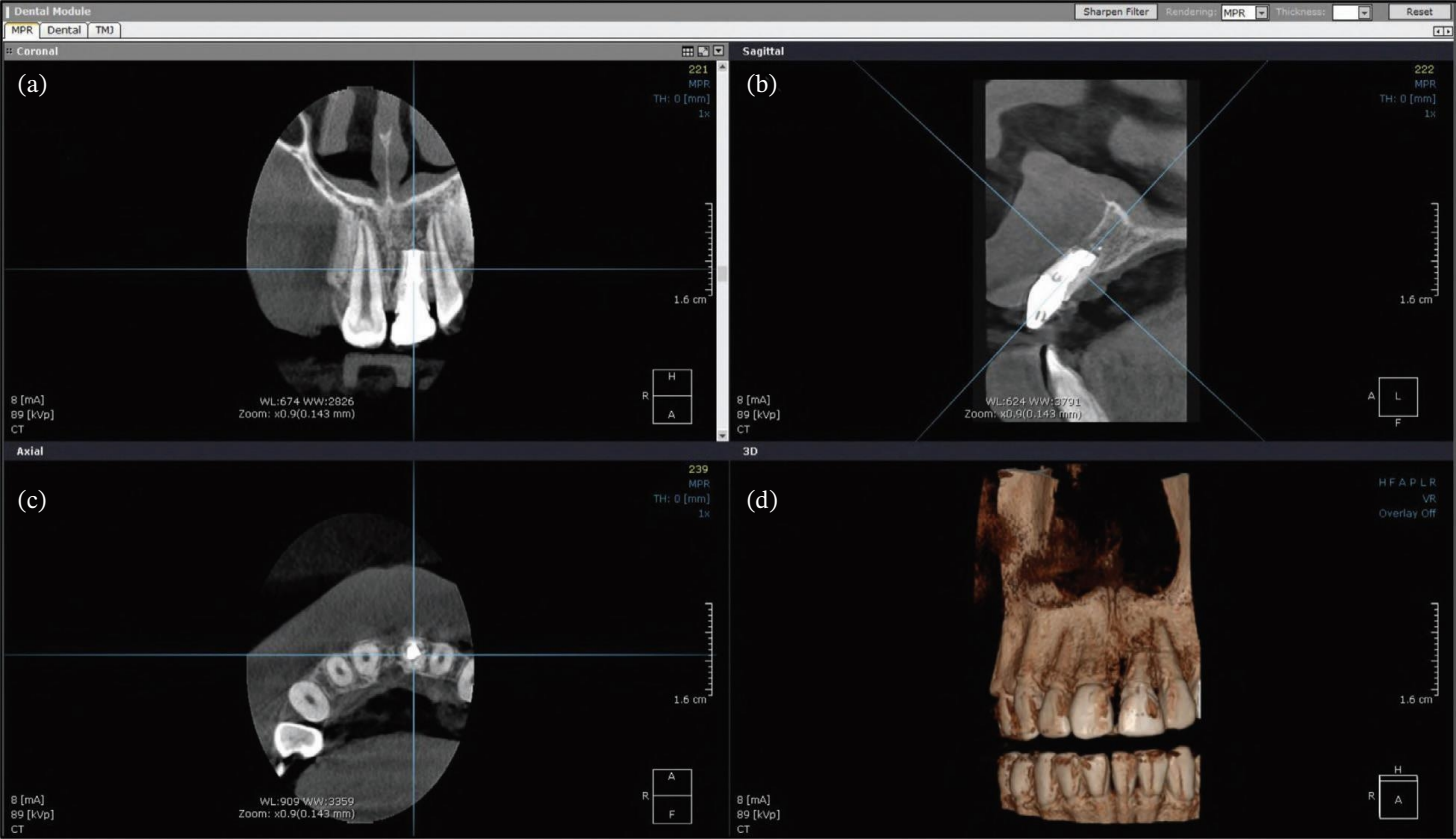
Follow‐up examination after surgical intervention by cone beam computed tomography. (a) Coronal, (b) sagittal, (c) axial, and (d) 3D reconstructions showing bone neoformation indicating repair in the periapical region.

The patient underwent a CBCT follow‐up 6 months after surgery, using the same parameters as before, on the EAGLE 3D tomograph (Dabi Atlante) at FORP/USP. The images were reconstructed using On‐Demand 3D software (Cybermed Inc.). The follow‐up examination is shown in Figure 5.

## 3. Discussion

Dental trauma is a common occurrence in sports, particularly in contact sports like football, basketball, martial arts, and hockey. Injuries to the mouth, teeth, and jaws can result from direct blows, falls, or collisions with equipment or other players. Therefore, sports dentistry plays a crucial role in managing dental trauma incidents among athletes [6, 7]. Prompt and specialized dental care is essential not only to restore the athlete′s oral health but also to minimize the impact on their performance and overall well‐being [6, 23]. Dentists specializing in sports dentistry are trained to assess and manage these traumatic injuries efficiently, often working in collaboration with sports medicine teams to ensure comprehensive care [6]. By focusing on prevention strategies, such as custom mouthguards [24], and providing immediate treatment when injuries occur [24], sports dentistry not only protects athletes′ smiles but also supports their ability to compete at their best.

CBCT was particularly valuable in the present case because it provided three‐dimensional information that supported diagnosis and surgical planning. This imaging modality allows detailed assessment of teeth, surrounding bone, and adjacent anatomical structures, thereby facilitating treatment planning in TDIs and their sequelae. Nonetheless, the indication for CBCT must always be properly justified, and the benefits of its use should outweigh the effects of ionizing radiation exposure. When indicated, it should follow the ALADA principles regarding radiation dose (“as low as diagnostically acceptable” and “as low as reasonably achievable”) [13, 14]. According to the American Association of Endodontists, CBCT should be considered the primary imaging tool of choice, in conjunction with clinical data, including chief complaint and radiographic findings, for planning periradicular surgeries to remove periapical lesions, locating root apices, and assessing proximity to adjacent anatomical structures [13, 14].

In the present case, the endodontic surgical approach was decisive for tooth preservation. Contemporary endodontic surgery, especially when performed with the aid of a surgical microscope and bioceramic materials, allows direct management of the periapical environment when conventional treatment alone is insufficient. Surgical intervention made it possible to eliminate the persistent extraradicular infection and to manage the lesion directly, supporting maintenance of the tooth in function within the stomatognathic system. This finding reinforces the role of surgical endodontics as a conservative and reliable alternative to extraction in selected cases.

The literature reports conflicting data regarding the prevalence ratio between periapical granulomas and cysts, with a review study indicating a higher prevalence of periapical cysts (0.03%–22.7%) compared to periapical granulomas (0.007%–17.3%) [25]. Periapical cysts are often associated with persistent periapical lesions following endodontic treatment. Conversely, other studies suggest that periapical granulomas are more frequent [26], with periapical cysts considered an evolutionary entity from granulomas. This underscores the importance of timing surgical intervention appropriately to resolve the case.

Regarding microbiological activity, the development of persistent periapical lesions is essentially linked to bacterial infection [27]. The bacterial genera *Fusobacterium*, *Parvimonas*, *Prevotella*, *Porphyromonas*, *Dialister*, *Streptococcus*, and *Treponema* are often associated with periapical lesions and found in polymicrobial communities [28]. In the present case, *P. aeruginosa* was identified in the clinical sample. Although uncommon in periapical lesions, this finding deserves attention, as opportunistic pathogens may contribute to infection persistence and therapeutic challenges. The identification of *P. aeruginosa* reinforces the importance of effective disinfection strategies, since persistent intraradicular infection is recognized as the main cause of posttreatment apical periodontitis [27].

In this case, different culture media and incubation conditions were employed in an attempt to recover a broader range of microorganisms potentially present in the infectious site. Nevertheless, only *P. aeruginosa* colonies were obtained. It is important to acknowledge that culture‐based methods may not detect fastidious microorganisms or bacteria present in low abundance. Since periapical infections are typically polymicrobial and molecular approaches have demonstrated greater microbial diversity than conventional culturing techniques [27], it is possible that additional microorganisms were present but not recovered under the conditions used in this study.

Despite the observed positive outcomes, this study has limitations as a single case report; the results cannot be generalized to a larger population, and the lack of a control group prevents a direct comparison of effectiveness against alternative treatments. However, this report remains valuable as a detailed clinical documentation that may guide future comparative studies. Finally, the protocol′s reliance on advanced technology, such as the surgical microscope and CBCT, may limit its applicability in resource‐limited clinical settings. Future studies with larger cohorts are encouraged to further validate these findings.

In conclusion, this case underscores the importance of multidisciplinary planning in the management of dental trauma, particularly when long‐term sequelae require restorative, endodontic, surgical, histopathological, and microbiological assessment. It also highlights the importance of adequate knowledge of initial emergency procedures, since early management may directly influence later treatment complexity and prognosis.

## Funding

This study was funded by the Coordenação de Aperfeiçoamento de Pessoal de Nível Superior (10.13039/501100002322) (33002029032P4), the Conselho Nacional de Desenvolvimento Científico e Tecnológico (10.13039/501100003593) (151773/2024‐6), and the Fundação de Amparo à Pesquisa do Estado de São Paulo (10.13039/501100001807) (2018/14450‐1 and 2021/01623‐8).

## Disclosure

Although the study received financial support from public funding agencies, these agencies had no role in the study design, data collection, analysis, interpretation of the results, or preparation and decision to publish the manuscript.

## Ethics Statement

Informed consent was obtained from the patient′s legal guardian (grandmother) for the treatment and publication of this case report. The study was approved by the institutional Research Ethics Committee (CAAE: 45617721.3.0000.5419) and was prepared in accordance with the Preferred Reporting Items for Case reports in Endodontics (PRICE) guidelines.

## Conflicts of Interest

The authors declare no conflicts of interest.

## Data Availability

Data sharing is not applicable to this article as no datasets were generated or analyzed during the current study.

## References

[1] Barrett E. J. and Kenny D. J. , Avulsed Permanent Teeth: A Review of the Literature and Treatment Guidelines, Dental Traumatology. (1997) 13, no. 4, 153–163, 10.1111/j.1600-9657.1997.tb00031.x, 9550040. 9550040

[2] Andreasen J. O. , Bakland L. K. , Matras R. C. , and Andreasen F. M. , Traumatic Intrusion of Permanent Teeth. Part 1. An Epidemiological Study of 216 Intruded Permanent Teeth, Dental Traumatology. (2006) 22, no. 2, 83–89, 10.1111/j.1600-9657.2006.00421.x, 2-s2.0-33644816142, 16499631. 16499631

[3] Sharif M. O. , Tejani-Sharif A. , Kenny K. , and Day P. F. , A Systematic Review of Outcome Measures Used in Clinical Trials of Treatment Interventions Following Traumatic Dental Injuries, Dental Traumatology. (2015) 31, no. 6, 422–428, 10.1111/edt.12227, 2-s2.0-84954388229, 26428411. 26428411

[4] Kenny K. P. , Day P. F. , Sharif M. O. , Parashos P. , Lauridsen E. , Feldens C. A. , Cohenca N. , Skapetis T. , Levin L. , Kenny D. J. , Djemal S. , Malmgren O. , Chen Y. J. , Tsukisboshi M. , and Andersson L. , What Are the Important Outcomes in Traumatic Dental Injuries? An International Approach to the Development of a Core Outcome Set, Dental Traumatology. (2018) 34, no. 1, 4–11, 10.1111/edt.12367, 2-s2.0-85034235310, 28873277. 28873277

[5] Lima T. F. R. , Dos Santos S. L. , da Silva Fidalgo T. K. , and Silva E. , Vitality Tests for Pulp Diagnosis of Traumatized Teeth: A Systematic Review, Journal of Endodontics. (2019) 45, no. 5, 490–499, 10.1016/j.joen.2019.01.014, 2-s2.0-85062698889, 30878165. 30878165

[6] Seth N. P. I. , Chaudhary M. , Kushwaha S. , and Sandhu K. S. , Sports Dentistry and Dental Traumatology- A Review, International Healthcare Research Journal. (2017) 1, no. 1, 2–6.

[7] Soares P. V. , Tolentino A. B. , Machado A. C. , Dias R. B. , and Coto N. P. , Sports Dentistry: A Perspective for the Future, Revista Brasileira de Educação Física e Esporte. (2014) 28, no. 2, 351–358, 10.1590/1807-55092014000200351.

[8] Barreiros D. , Nelson P. F. , Paula-Silva F. W. G. , Oliveira K. M. H. , Lucisano M. P. , Rossi A. , Silva L. A. B. , Küchler E. C. , and Silva R. A. B. , MMP2 and MMP9 Are Associated With Apical Periodontitis Progression and Might Be Modulated by TLR2 and MyD88, Brazilian Dental Journal. (2018) 29, no. 1, 43–47, 10.1590/0103-6440201801731, 2-s2.0-85038421936, 29267523. 29267523

[9] Mazzi-Chaves J. F. , Petean I. B. F. , Soares I. M. V. , Salles A. G. , Antunes L. A. A. , Segato R. A. B. , Silva L. , Küchler E. C. , Antunes L. S. , and Sousa-Neto M. D. , Influence of Genetic Polymorphisms in Genes of Bone Remodeling and Angiogenesis Process in the Apical Periodontitis, Brazilian Dental Journal. (2018) 29, no. 2, 179–183, 10.1590/0103-6440201802260, 2-s2.0-85048589819, 29898065. 29898065

[10] Márton I. J. and Kiss C. , Overlapping Protective and Destructive Regulatory Pathways in Apical Periodontitis, Journal of Endodontics. (2014) 40, no. 2, 155–163, 10.1016/j.joen.2013.10.036, 2-s2.0-84892979129, 24461396. 24461396

[11] Romualdo P. C. , Lucisano M. P. , Paula-Silva F. W. G. , Leoni G. B. , Sousa-Neto M. D. , Silva R. A. B. , Silva L. A. B. , and Nelson-Filho P. , Ovariectomy Exacerbates Apical Periodontitis in Rats With an Increase in Expression of Proinflammatory Cytokines and Matrix Metalloproteinases, Journal of Endodontics. (2018) 44, no. 5, 780–785, 10.1016/j.joen.2018.01.010, 2-s2.0-85043536730, 29550006. 29550006

[12] Dessaune Neto N. , Porpino M. T. M. , Antunes H. D. S. , Rodrigues R. C. V. , Perez A. R. , Pires F. R. , Siqueira J. F. , and Armada L. , Pro-Inflammatory and Anti-Inflammatory Cytokine Expression in Post-Treatment Apical Periodontitis, Journal of Applied Oral Science. (2018) 26, 10.1590/1678-7757-2017-0455, 2-s2.0-85050443116, 29898177. PMC5963913 29898177

[13] Mazzi-Chaves J. F. , Camargo R. V. , Borges A. F. , Silva R. G. , Pauwels R. , Silva-Sousa Y. T. C. , and Sousa-Neto M. D. , Cone-Beam Computed Tomography in Endodontics—State of the Art, Current Oral Health Reports. (2021) 8, no. 2, 9–22, 10.1007/s40496-021-00292-8.

[14] Maia Filho E. M. , Calisto A. M. , De Jesus Tavarez R. R. , de Castro Rizzi C. , Bezerra Segato R. A. , and Bezerra da Silva L. A. , Correlation Between the Periapical Index and Lesion Volume in Cone-Beam Computed Tomography Images, Iranian Endodontic Journal. (2018) 13, no. 2, 155–158, 10.22037/iej.v13i2.15040, 2-s2.0-85045130196, 29707007. 29707007 PMC5911286

[15] Nagendrababu V. , Chong B. S. , McCabe P. , Shah P. K. , Priya E. , Jayaraman J. , Pulikkotil S. J. , and Dummer P. M. H. , Guidelines for Reporting the Quality of Clinical Case Reports in Endodontics: A Development Protocol, International Endodontic Journal. (2019) 52, no. 6, 775–778, 10.1111/iej.13067, 2-s2.0-85060510831, 30586165. 30586165

[16] Venskutonis T. , Plotino G. , Tocci L. , Gambarini G. , Maminskas J. , and Juodzbalys G. , Periapical and Endodontic Status Scale Based on Periapical Bone Lesions and Endodontic Treatment Quality Evaluation Using Cone-Beam Computed Tomography, Journal of Endodontics. (2015) 41, no. 2, 190–196, 10.1016/j.joen.2014.10.017, 2-s2.0-84921543019, 25498834. 25498834

[17] Versiani M. A. , Pécora J. D. , and Sousa-Neto M. D. , Microcomputed Tomography Analysis of the Root Canal Morphology of Single-Rooted Mandibular Canines, International Endodontic Journal. (2013) 46, no. 9, 800–807, 10.1111/iej.12061, 2-s2.0-84881548478, 23402296. 23402296

[18] Boschetti E. , Silva-Sousa Y. T. C. , Mazzi-Chaves J. F. , Leoni G. B. , Versiani M. A. , Pécora J. D. , Saquy P. C. , and Sousa-Neto M. D. , Micro-CT Evaluation of Root and Canal Morphology of Mandibular First Premolars With Radicular Grooves, Brazilian Dental Journal. (2017) 28, no. 5, 597–603, 10.1590/0103-6440201601784, 2-s2.0-85035145581, 29215685. 29215685

[19] Bornstein M. M. , Bingisser A. C. , Reichart P. A. , Sendi P. , Bosshardt D. D. , and von Arx T. , Comparison Between Radiographic (2-Dimensional and 3-Dimensional) and Histologic Findings of Periapical Lesions Treated With Apical Surgery, Journal of Endodontics. (2015) 41, no. 6, 804–811, 10.1016/j.joen.2015.01.015, 2-s2.0-84930085161, 25863407. 25863407

[20] Murray C. A. and Saunders W. P. , Root Canal Treatment and General Health: A Review of the Literature, International Endodontic Journal. (2000) 33, no. 1, 1–18, 10.1046/j.1365-2591.2000.00293.x, 2-s2.0-0033766401. 11307468

[21] McElvania Tekippe E. , Shuey S. , Winkler D. W. , Butler M. A. , and Burnham C. A. , Optimizing Identification of Clinically Relevant Gram-Positive Organisms by Use of the Bruker Biotyper Matrix-Assisted Laser Desorption Ionization-Time of Flight Mass Spectrometry System, Journal of Clinical Microbiology. (2013) 51, no. 5, 1421–1427, 10.1128/jcm.02680-12, 2-s2.0-84876765761, 23426925. 23426925 PMC3647943

[22] Tewari N. , O′Connell A. C. , Abbott P. V. , Mills S. C. , Stasiuk H. , Roettger M. , and Levin L. , The International Association of Dental Traumatology (IADT) and the Academy for Sports Dentistry (ASD) Guidelines for the Prevention of Traumatic Dental Injuries: Part 9: Role of Dental Professionals, Dental Traumatology. (2024) 40, no. Suppl 1, 20–21, 10.1111/edt.12930, 38363706. 38363706

[23] Abbott P. V. , Tewari N. , O′Connell A. C. , Mills S. C. , Stasiuk H. , Roettger M. , and Levin L. , The International Association of Dental Traumatology (IADT) and the Academy for Sports Dentistry (ASD) Guidelines for Prevention of Traumatic Dental Injuries: Part 3: Mouthguards for the Prevention of Dental and Oral Trauma, Dental Traumatology. (2024) 40, 7–9, 10.1111/edt.12925, 38363704. 38363704

[24] Couto A. M. D. , Meirelles D. P. , Valeriano A. T. , Almeida D. S. , Moraes Ê. , Tarquinio S. B. C. , Batista A. C. , MendonÇa E. F. , Costa N. D. L. , Alves P. M. , Nonaka C. F. W. , Abreu L. G. , and Aguiar M. C. F. , Chronic Inflammatory Periapical Diseases: A Brazilian Multicenter Study of 10,381 Cases and Literature Review, Brazilian Oral Research. (2021) 35, 10.1590/1807-3107bor-2021.vol35.0033. 33729278

[25] Sullivan M. , Gallagher G. , and Noonan V. , The Root of the problem, Journal of the American Dental Association. (2016) 147, no. 8, 646–649, 10.1016/j.adaj.2016.02.018, 2-s2.0-84961942958. 27046538

[26] Siqueira J. F. , Rôças I. N. , Ricucci D. , and Hülsmann M. , Causes and Management of Post-Treatment Apical Periodontitis, British Dental Journal. (2014) 216, no. 6, 305–312, 10.1038/sj.bdj.2014.200, 2-s2.0-84896961475, 24651336. 24651336

[27] Horiuchi A. , Kokubu E. , Warita T. , and Ishihara K. , Synergistic Biofilm Formation by Parvimonas micra and Fusobacterium nucleatum, Anaerobe. (2020) 62, 102100, 10.1016/j.anaerobe.2019.102100, 2-s2.0-85072397712, 31521732. 31521732

[28] Ricucci D. and Siqueira J. F. , Biofilms and Apical Periodontitis: Study of Prevalence and Association With Clinical and Histopathologic Findings, Journal of Endodontics. (2010) 36, no. 8, 1277–1288, 10.1016/j.joen.2010.04.007, 2-s2.0-77955423520. 20647081

